# Thermomechanical Comparison of PET-G and TC-85 Orthodontic Aligner Materials

**DOI:** 10.3390/polym18202517

**Published:** 2026-10-14

**Authors:** Melis Büşra Aşkın, Serkan Görgülü, Gökhan Serhat Duran

**Affiliations:** 1 Department of Orthodontics, Faculty of Dentistry, Yozgat Bozok University, 66100 Yozgat, Türkiye; 2 Department of Orthodontics, Gülhane Faculty of Dental Medicine, University of Health Sciences, 06010 Ankara, Türkiye; serkan.gorgulu@sbu.edu.tr; 3 Department of Orthodontics, Faculty of Dentistry, Çanakkale Onsekiz Mart University, 17100 Canakkale, Türkiye; gokhanserhat.duran@comu.edu.tr

**Keywords:** clear aligners, PET-G, TC-85, thermomechanical characterisation, finite element analysis, shape recovery, dynamic mechanical analysis

## Abstract

This study combined thermomechanical characterisation with finite element analysis to compare polyethylene terephthalate glycol (PET-G) and Tera Harz TC-85 orthodontic aligner materials. Tensile properties were evaluated at 23, 37, 55 and 80 °C, with five observations per material and temperature. Identical full-arch geometries with a nominal thickness of 0.75 mm were subjected to 600 N compression at 300 K, heating under maintained load to 352 K and cooling to 310 K. At 23 °C, tensile moduli were 1396.4 ± 44.9 MPa for PET-G and 675.4 ± 21.2 MPa for TC-85. Dynamic mechanical analysis (DMA) loss-tangent maxima occurred at sampled temperatures of 80 and 45 °C. Tensile and DMA moduli diverged at elevated temperatures. Initial maximum effective strains were 0.165 and 0.196, and medium-to-fine mesh changes were 1.78% and 2.61%. TC-85 showed a greater reduction in maximum strain following heating to 78.85 °C. Recovery benchmark values at 60 min were 63.3% for PET-G and 92.5% for TC-85; paired-series metrics quantified curve agreement. PET-G exhibited higher tensile stiffness, whereas TC-85 exhibited greater benchmark recovery and a larger thermal-stage strain reduction. The high-temperature endpoint represents ex vivo activation. Constitutive and experimental limitations constrain extrapolation to clinical appliance performance.

## 1. Introduction

Clear aligners deform when seated on the dentition, and their mechanical response changes during wear. Polymer composition, fabrication history and environmental exposure influence stiffness and force relaxation. Thermoforming can also alter the thickness and thermal properties of an appliance [1]. Direct printing produces a different combination of material structure and geometry, with dimensional accuracy affected by printing and post-curing conditions [2,3]. A material comparison therefore requires both mechanical characterisation and a clear account of the geometry and conditions used in the analysis.

PET-G is a thermoplastic copolyester widely used for thermoformed appliances. TC-85 is a commercial photocurable resin used for direct-printed aligners. Experimental studies have examined its viscoelastic response and thermally activated shape recovery [4,5,6]. Reported transition temperatures depend on the measurement method and specimen history. Lee et al. evaluated recovery following thermal programming and distinguished dynamic mechanical properties from static tensile behaviour [4]. Atta et al. characterised the material using thermal and mechanical measurements [5]. Choi et al. examined temperature-dependent stiffness and viscoelasticity over an oral-temperature range [6]. These studies support evaluating temperature and loading time explicitly.

Finite element analysis provides a way to compare material assignments within a common geometry. A previous study examined aligner materials under compressive loading up to 600 N [7]. Such an imposed appliance load differs from the force transmitted to an individual tooth. For a temperature-driven comparison, the material law must also describe changes in stiffness and time dependence over the temperature range being considered. Agreement with a recovery curve used during calibration measures the fit of that curve; predictive performance requires a separate experimental comparison.

Temperature near 37 °C represents oral service, while 45 °C provides a comparison under transient warming. A temperature of 78.85 °C represents an ex vivo activation condition. The position of each temperature relative to the material transition is relevant because heating can change both stiffness and recovery. Wet exposure adds another source of variation: the effect of immersion on relaxation depends on the material and test conditions [8].

This study combined experimental material characterisation with a finite element comparison of PET-G and TC-85 under a common geometry and thermomechanical loading sequence. The primary numerical outcomes were maximum effective strain during loading and at the heating and cooling endpoints. Tensile, thermal and recovery measurements provided material-level context for the comparison and informed the reference material assignments. The hypothesis was that TC-85 would exhibit greater initial deformation but a larger temperature-associated reduction in strain than PET-G. Initial stiffness, recovery fraction and thermal-stage changes in the aligner strain field were evaluated as distinct outcomes.

## 2. Materials and Methods

### 2.1. Study Design and Experimental Characterisation

Transparent DURAN PET-G sheets (SCHEU-DENTAL, Iserlohn, Germany) and Tera Harz TC-85DAC resin (Graphy Inc., Seoul, Republic of Korea; hereafter TC-85) were investigated by thermomechanical characterisation and full-arch finite element analysis. PET-G coupons were cut from flat sheets, and TC-85 coupons were printed at the same nominal thickness of 1.0 mm. One material lot was used for each polymer. The specimen thickness was distinct from the 0.75 mm nominal CAD aligner wall. Characterisation comprised uniaxial tension, differential scanning calorimetry (DSC), dynamic mechanical analysis (DMA), stress relaxation, creep–recovery, three-point bending and Vickers microhardness.

TC-85 specimens were fabricated with a Uniz 4K printer(UNIZ Technology, Beijing, China) using 100 µm layers, 1.3 s exposure per layer and a vertical build orientation of 90° to the build platform. Residual resin was removed with a soft scraper and fine brush. Post-curing was performed for 20 min in a Tera Harz Cure unit under nitrogen using 385–405 nm UV light.

After cutting or post-curing, specimens were conditioned for 48 h at 23 ± 2 °C and 50 ± 5% relative humidity. Dimensions were measured at three positions with a calibrated micrometre. Before temperature-chamber testing, specimens were equilibrated at the target temperature for 10 min, with a tolerance of ±0.5 °C.

Tensile tests used rectangular coupons measuring 60 × 10 × 1.0 mm, an initial grip separation of 40 mm and an optical gauge length of 30 mm. The crosshead speed was 1 mm/min over the modulus interval and 5 mm/min thereafter to fracture. Young’s modulus was evaluated over 0.05–0.25% axial strain. Tensile modulus, ultimate tensile strength and elongation were evaluated at 23, 37, 55 and 80 °C, with five observations per material and temperature. Table 1 presents tensile modulus; Supplementary Table S1 includes strength and elongation.

DMA was performed with a TA Instruments Q800(TA Instruments, New Castle, DE, USA) in tension mode using 40 × 5 × 1.0 mm coupons and a 10 mm free gauge length. The oscillation frequency was 1 Hz, the strain amplitude was 0.1%, and temperature increased from 20 to 100 °C at 5 °C/min. Storage modulus E′ and loss-tangent tan δ were evaluated.

DSC was performed with a TA Instruments Q2000 (TA Instruments, New Castle, DE, USA) using 5.0 ± 0.5 mg specimens in aluminium pans under nitrogen at 50 mL/min. The temperature programme was 0→150→0→150 °C, with heating and cooling rates of 10 °C/min and 3 min holds.

The thermal series was reported over 20–100 °C at 5 °C intervals. The DMA peak temperature was the sampled temperature with the highest tan δ. Early stiffness loss was described by the first interval in which E′ fell below 95% of its 20 °C value. The DSC rise was described by the adjacent interval with the largest positive signal increment, expressed in arbitrary units. Nominal transition estimates of 78 and 43 °C for PET-G and TC-85 were retained as approximate thermal references, separately from these sampled descriptors. At 23 and 37 °C, E′ was linearly interpolated between neighbouring temperatures for comparison with tensile modulus. The 5 °C reporting interval was distinct from the DMA heating rate of 5 °C/min and the DSC heating rate of 10 °C/min.

Stress relaxation was evaluated at 37 °C using the tensile-coupon geometry. Axial strain was increased to 1.0% over 10 s and held for 60 min. Force was recorded at 10 Hz during loading and the first minute, then at 1 Hz. Creep tests used rectangular tensile coupons at 37 °C under a constant stress of 1.0 MPa for 60 min, followed by unloading and 60 min of strain-recovery monitoring.

Shape-recovery specimens were 50 × 10 × 1.0 mm strips bent around a 4 mm diameter mandrel at 80 °C and held for 5 min. They were cooled under constraint to 24 °C, held for 5 min and fully unloaded. Free recovery was monitored by video for 60 min in an 80 °C water bath. Benchmark and model recovery fractions at 0, 5, 10, 20, 30, 45 and 60 min were compared using all seven pairs at full numerical precision. Recovery fraction described this free-recovery series, whereas reduction in maximum aligner strain compared numerical field maxima under maintained compression. The paired-series comparison quantified curve agreement.

Three-point bending used 30 × 10 × 1.0 mm strips, a 16 mm support span and a crosshead speed of 1 mm/min at 23, 37 and 55 °C. Vickers microhardness was measured on 10 × 10 × 1.0 mm flat coupons at 23 °C using a Qness 60 M Evo (ATM Qness GmbH, Golling, Austria) with a 1.96 N load and 10 s dwell time (HV0.2). Five indentations were averaged for each material group. Supplementary Tables S6 and S8 provide the flexural and hardness summaries, and Supplementary Data S1 contains the numerical values and calculation procedures.

### 2.2. Geometry and Loading Sequence

A digital dental model in stereolithography (STL) format was imported into Maestro 3D Dental Studio Expert (version 7.00.00.30000, Ortho Studio module; AGE Solutions S.r.l., Pontedera, Italy). A full-arch aligner was generated with a uniform nominal CAD wall thickness of 0.75 mm. The aligner surface was offset from the dental crowns to define the intaglio fit. The same geometry and homogeneous, isotropic material assumptions were used for PET-G and TC-85. The nominal CAD thickness describes the computational geometry; it is not a measurement of a fabricated appliance.

Finite-sliding, separable surface-to-surface contact was defined between the aligner intaglio surface and the dental crowns. The Coulomb static and dynamic friction coefficients were μs = μd = 0.20. A 2 mm posterior support band in the terminal molar regions constrained the posterior occlusal and lingual aligner surfaces, leaving the anterior segment free to deform. The load was distributed over the labial surface of the central-incisor region, with a resultant of (0, 0, −600) N in the model coordinate system. This 600 N condition provides a high-load appliance comparison associated with biting-load studies [7]; it does not represent force on an individual tooth.

In Ansys LS-DYNA R12.0.0 (Ansys, Inc., Canonsburg, PA, USA), the compressive load increased smoothly from 0 to 600 N over 10 s at 300 K (26.85 °C), followed by a 50 s hold. With compression maintained, temperature increased to 352 K (78.85 °C) over 600 s, remained at 352 K for 600 s, decreased to 310 K (36.85 °C) over 600 s and remained at 310 K for a final 600 s. Supplementary Table S10 gives the cumulative timing. The 352 K stage represents ex vivo activation. The reported numerical outcomes are the initial, heating and cooling stage endpoints.

### 2.3. Material Assignments and Links to Characterisation

The reference elastic moduli assigned to PET-G and TC-85 were 1396 and 675 MPa, respectively, informed by room-temperature tensile characterisation. The reference Poisson ratios were 0.36 and 0.40, and densities were 1.27 and 1.15 g cm^−3^. Constant thermal expansion coefficients of 6.5 × 10^−5^ and 1.0 × 10^−4^ K^−1^ were specified relative to 300 K. Density, Poisson ratio and thermal expansion were assigned model inputs. Supplementary Table S7 lists the reference properties, while Table 1 reports experimental tensile means and sample standard deviations.

PET-G was represented by finite-strain thermoviscoelasticity. Its specified shear-relaxation fraction was *g*_1_ = 0.55, with an equilibrium fraction of 0.45 and an Arrhenius activation energy of 50 kJ/mol. TC-85 used *MAT_291 (*MAT_SHAPE_MEMORY_ALLOY) with symmetric isotropic transformation surfaces. The additional model coefficients are given in Supplementary Table S9. MAT_291 was formulated for alloy transformation [9]; its branch labels are used phenomenologically here and do not identify physical martensitic or austenitic phases in TC-85. Table 2 relates the characterisation datasets to the material comparison. Supplementary Table S5 gives the separate algebraic relaxation, creep and recovery functions with their coefficients and time units.

### 2.4. Mesh Refinement and Numerical Outcomes

The full-arch aligner was discretised with 10-node quadratic tetrahedral elements (ELFORM 16), with at least three element layers through the thickness. Mesh quality was assessed by aspect ratio: most elements were below 10 and the maximum was 11.23. Coarse, medium and fine meshes contained 119,369, 296,750 and 671,671 elements, respectively. Maximum effective strain at 600 N and 300 K was compared across these meshes, and the fine mesh was used for the full-arch results. Relative change was calculated as 100 × |*ε*_fine_ − *ε*_coarse_|/|*ε*_fine_|, using the next-finer result as the denominator. The 5% criterion was applied specifically to initial peak strain.

The analysis used nonlinear implicit dynamics with an initial time step of 0.01 s and a minimum of 10^−4^ s. Upper time-step limits ranged from 0.5 to 5 s. The Newmark parameters were γ = 0.55 and β = 0.275625. These solver controls accompanied the prescribed load and temperature sequence.

Maximum effective strain was compared across initial loading, the heating endpoint and the cooling endpoint. Displacement in millimetres and equivalent stress in megapascals were assessed for the initial and heating endpoints. Stress and strain were evaluated as separate field maxima. Estimation of modulus from their ratio would require matching location, time, strain measure and constitutive state.

Reduction in maximum strain was calculated as 100 × (*ε*_initial_ − *ε*_stage_)/*ε*_initial_. This quantity describes the change in field maxima across thermal stages under the stated loading protocol. Numerical endpoints were treated as deterministic quantities, separately from variability in material-property records.

### 2.5. Descriptive Analysis

Tensile properties are presented as mean ± sample SD, using the five observations per condition and the n − 1 denominator. Modulus loss from 23 to 80 °C was calculated relative to the 23 °C mean. The difference between tensile modulus E and DMA storage modulus E′ was calculated as 100 × (E′ − E)/E′. This comparison aligns nominal temperature while retaining the distinct tensile loading rate and DMA frequency.

Recovery agreement was calculated from all seven pairs in Supplementary Table S4, including zero time. For model fraction ŷ_i_ and benchmark fraction y_i_, MAE = Σ|ŷ_i_ − y_i_|/n and RMSE = [Σ(*ŷ*_i_ − *y*_i_)^2^/n]^1^ᐟ^2^, with n = 7. Both metrics are expressed in recovery-fraction units; multiplying by 100 gives percentage points. Calculations use full numerical precision, with rounding applied for display. MAE and RMSE quantify descriptive agreement between the paired series.

## 3. Results

### 3.1. Tensile Stiffness and Thermal Characterisation

PET-G had the higher tensile modulus at each temperature. From 23 to 80 °C, its mean modulus decreased from 1396.4 to 503.3 MPa (64.0%). TC-85 decreased from 675.4 to 127.8 MPa (81.1%). At 37 °C, the corresponding means were 1078.4 and 463.7 MPa. TC-85 had greater elongation at all four temperatures (Supplementary Table S1).

The largest sampled DSC signal increments occurred over 75–80 °C for PET-G and 40–45 °C for TC-85. The tan δ maxima occurred at 80 and 45 °C (Supplementary Figure S1). The first 5% decrease in E′ from the 20 °C reference occurred within 60–65 °C and 25–30 °C, respectively. These descriptors retain the 5 °C sampling resolution and represent distinct thermal features.

**Figure 1:**
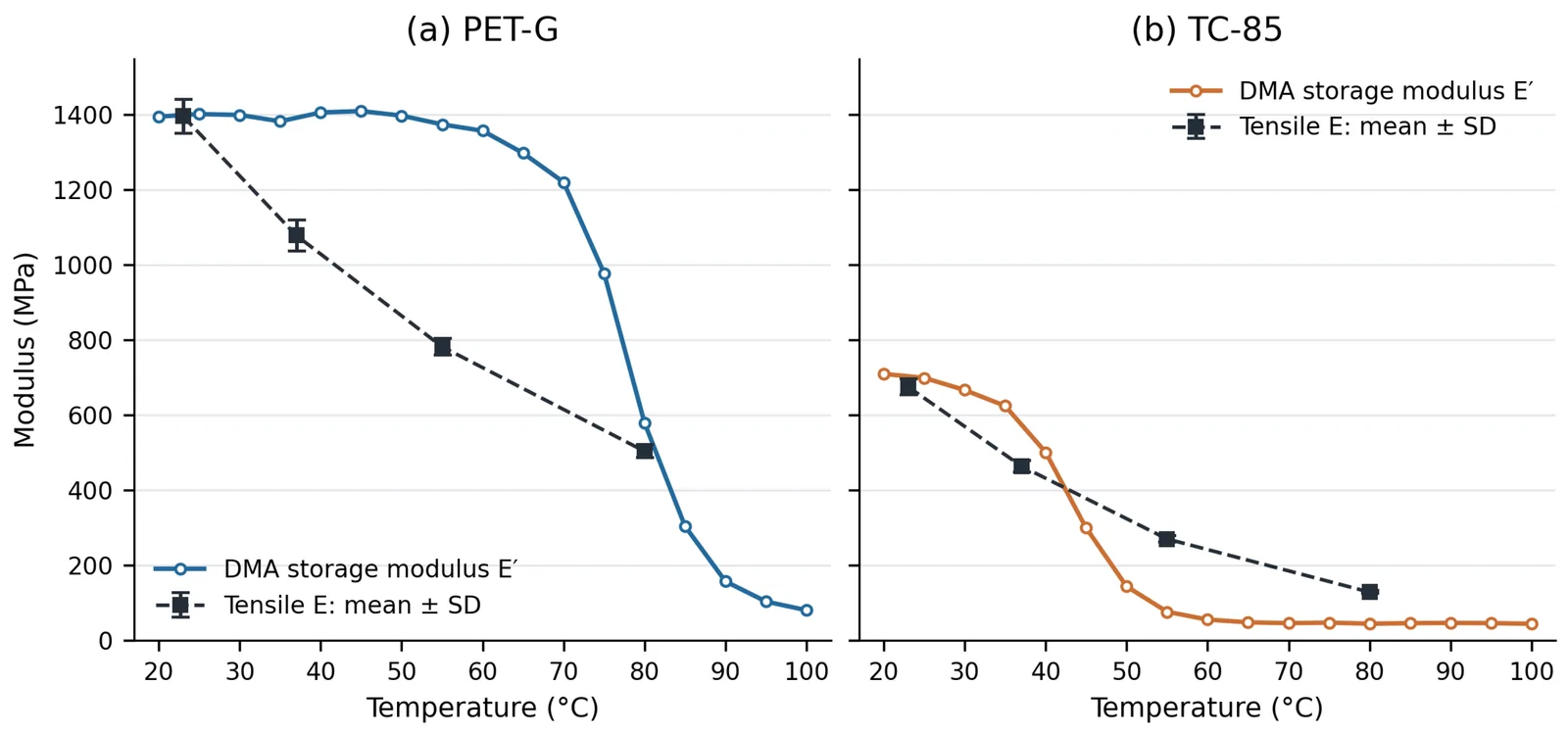
Temperature-dependent stiffness of (**a**) PET-G and (**b**) TC-85. Each panel compares DMA storage modulus at 5 °C reporting intervals with tensile means ± sample SD from five observations per condition. Lines are visual guides. Moduli are compared at common nominal temperatures.

The tensile and DMA series diverged at elevated temperatures (Figure 1 and Supplementary Table S2). At 55 °C, PET-G had E = 781.9 MPa and E′ = 1374.5 MPa; E was 43.1% below E′. For TC-85, E = 269.5 MPa and E′ = 75.4 MPa. At 37 °C, interpolated E′ values were 1392.1 and 574.8 MPa; the corresponding tensile means were 22.5% and 19.3% lower. The reversal of the TC-85 difference at higher temperatures prevents reconciliation by a single common correction factor.

### 3.2. Initial Numerical Response and Mesh Refinement

At the 600 N initial condition, maximum effective strain was 0.165 for PET-G and 0.196 for TC-85 (Table 3); the TC-85 value was 18.7% higher. Maximum displacements were 0.1319 and 0.1565 mm, and equivalent-stress maxima were 80.8 and 47.9 MPa. The 23 °C tensile strengths were 54.8 ± 1.5 and 35.0 ± 0.8 MPa.

Coarse-to-medium changes in peak strain were 3.79% for PET-G and 5.04% for TC-85. The latter exceeded the 5% criterion. Medium-to-fine changes were 1.78% and 2.61%, below that threshold for initial peak strain. Supplementary Table S3 provides the underlying values.

### 3.3. Thermal-Stage Response

**Figure 2:**
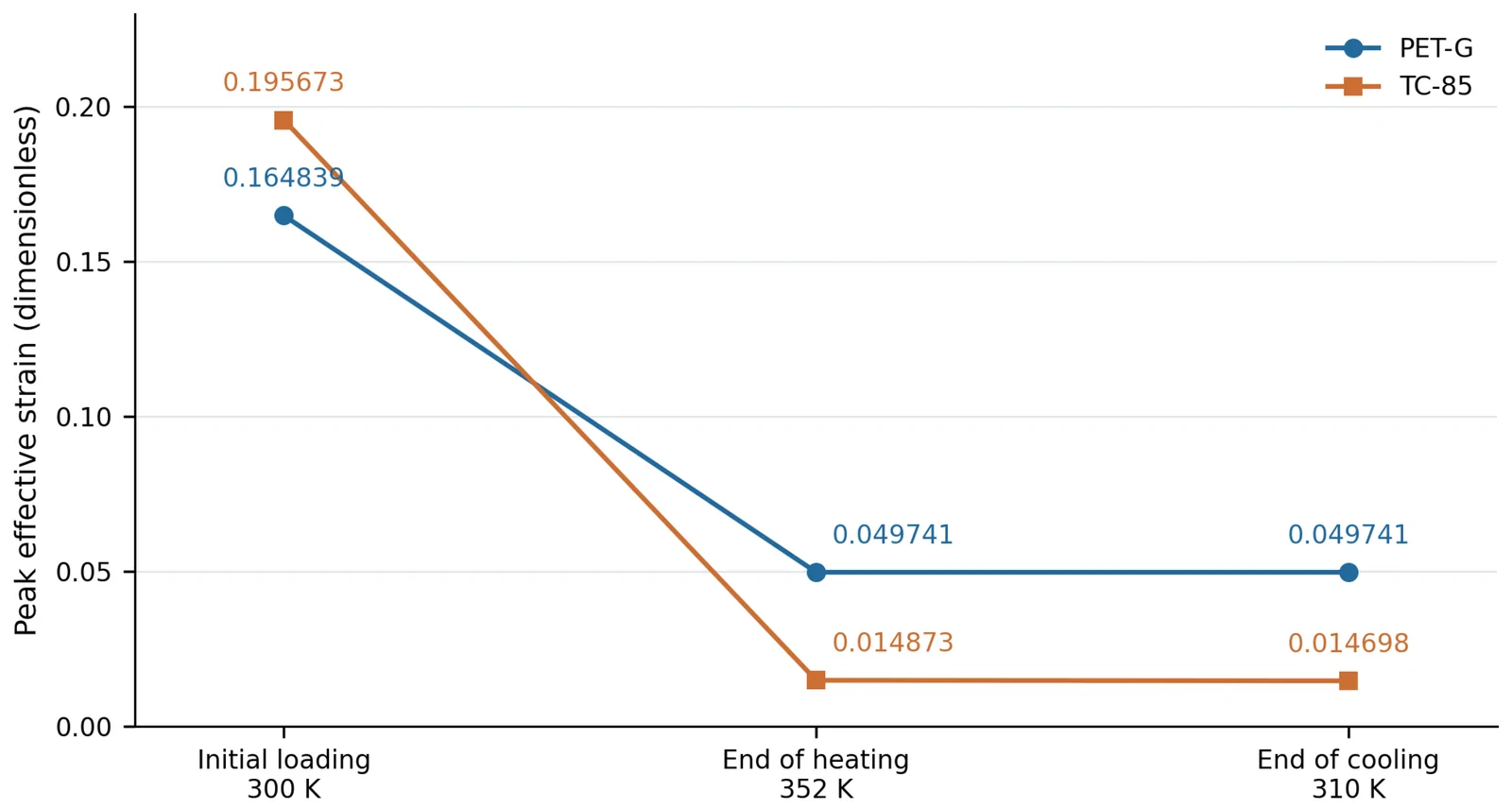
The maximum effective strain at the three stages of the thermomechanical loading sequence. The horizontal position denotes stage rather than elapsed time. Heating to 352 K represents ex vivo activation; the final 310 K state follows this thermal history.

Heating-end strains at 352 K were 0.049741 for PET-G and 0.014873 for TC-85, corresponding to reductions of 69.8% and 92.4% from the initial maxima. Cooling-end strains were 0.049741 and 0.014698. PET-G heating and cooling values were indistinguishable at six-decimal reporting precision. TC-85 strain decreased by 1.18% relative to its heating-end value. Figure 2 presents the three discrete stages.

### 3.4. Agreement Between the Recovery Series

**Figure 3:**
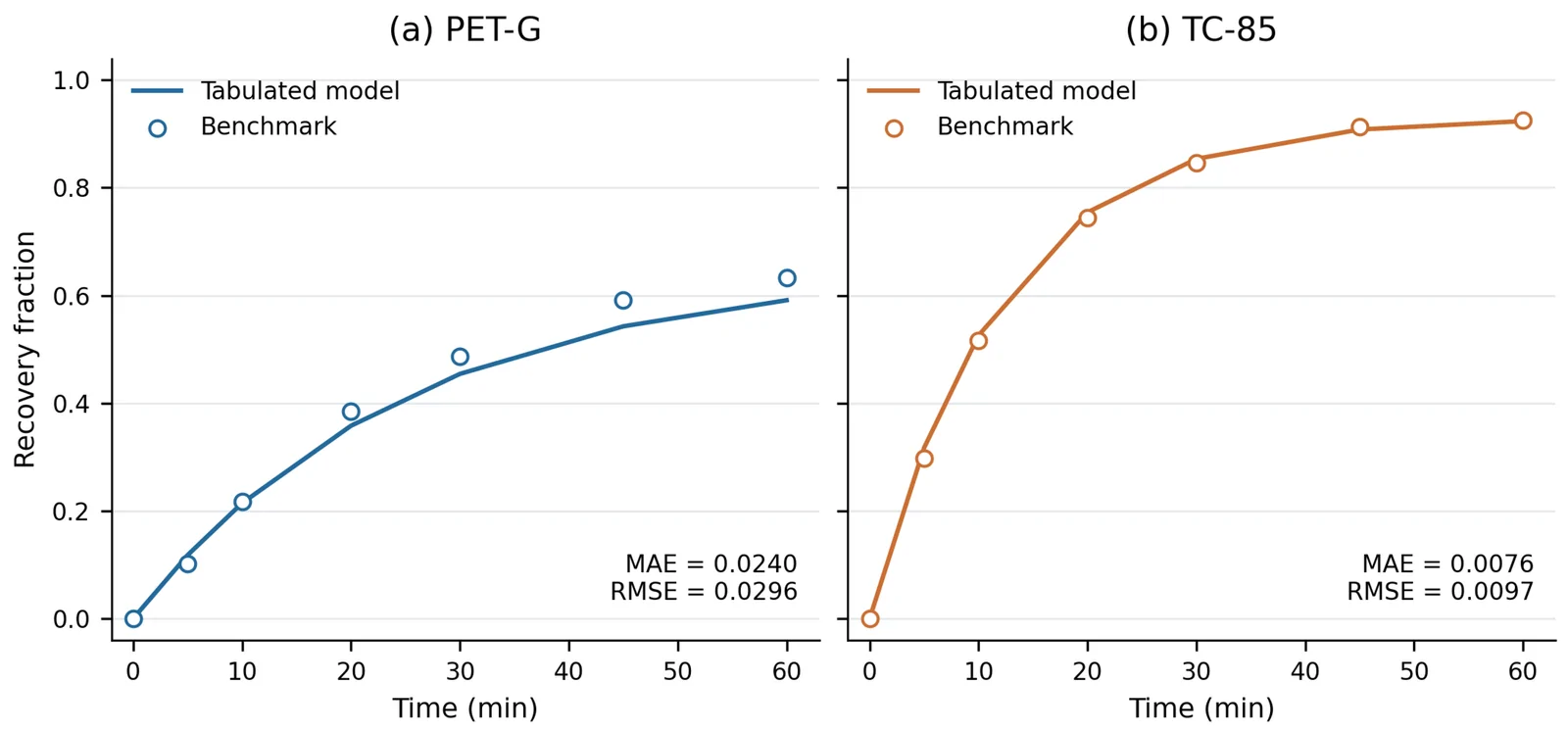
The agreement between recovery benchmark and model series. The markers show benchmark fractions, and the lines join model fractions at the same times. MAE and RMSE describe the seven paired values.

Benchmark recovery at 60 min was 0.6333 for PET-G and 0.9254 for TC-85. Corresponding model fractions were 0.5910 and 0.9237. Across the seven pairs, MAE was 0.0240 and 0.0076, and RMSE was 0.0296 and 0.0097, respectively. In percentage points, MAE was 2.40 and 0.76, and RMSE was 2.96 and 0.97. Figure 3 shows the paired series and Supplementary Table S4 provides the values.

## 4. Discussion

### 4.1. Stiffness and Recovery Describe Different Responses

PET-G exhibited higher tensile stiffness, whereas TC-85 exhibited greater benchmark recovery. The lower initial numerical strain of PET-G is consistent with its higher reference stiffness. Orthodontic force delivery also depends on seating displacement, appliance fit and supporting-tissue stiffness. The initial strain ranking therefore describes deformation under the prescribed load, while clinical force systems depend on the wider appliance–tooth interaction.

Temperature-responsive recovery in TC-85 has been examined experimentally [4,5,6]. The present free-recovery measurements at 80 °C followed programming, constrained cooling and unloading. This protocol differs mechanically from heating an aligner under maintained compression. Recovery fraction and thermal-stage strain reduction are therefore interpreted as distinct outcomes. The benchmark informed the recovery response, so the paired-series metrics quantify curve agreement. Independent material and full-arch comparisons are needed to establish predictive validity.

### 4.2. Temperature Dependence and the DMA–Tensile Comparison

The difference between tensile modulus and E′ is central to interpreting temperature-dependent behaviour. DMA used a 1 Hz oscillation at 0.1% strain amplitude, whereas tensile modulus was evaluated over 0.05–0.25% axial strain at a crosshead speed of 1 mm/min. These different loading conditions can contribute to differences between the moduli. Temperature equilibration, strain measurement, grip compliance, thermal expansion and specimen preparation may also contribute. Their individual effects were not isolated. For TC-85, tensile modulus exceeds E′ at higher temperatures, reversing the relationship near room temperature.

Constitutive calibration links stiffness, relaxation and recovery to loading and thermal history. The reversal of the TC-85 tensile–DMA difference shows why these quantities should retain their measurement-specific interpretation. Tensile and flexural moduli describe different loading configurations, while the algebraic functions in Supplementary Table S5 describe the time dependence of the associated curves.

The sampled DSC rise, tan δ maximum and first 5% loss in E′ describe different thermal features. The DSC rise is expressed as an interval in an arbitrary-unit signal; it is distinct from an instrument-defined onset or midpoint Tg. The nominal 78 and 43 °C estimates serve as approximate thermal references. The 5 °C reporting grid limits the resolution of peak identification. These descriptors should be kept separate when interpreting behaviour around 37–45 °C and at the activation temperature.

### 4.3. Interpretation of the Numerical Thermal Endpoints

The 352 K endpoint corresponds to 78.85 °C, approximately 1 and 36 °C above the nominal transition estimates of 78 and 43 °C for PET-G and TC-85, respectively. Equal absolute temperature therefore places the two materials at different positions relative to their transitions. The final 310 K state reflects the preceding activation history and cannot establish the response to a separate isothermal 37 °C exposure. A comparison at matched offsets from consistently defined transition markers would address a different thermal condition.

Under maintained load, reduced modulus alone would not explain the observed PET-G strain reduction. Thermal strain, geometry, constraints and constitutive internal variables can also affect a field maximum. The thermal-stage change is therefore interpreted as a combined model response, without assigning it to a single recovery mechanism. The PET-G endpoints are effectively unchanged during cooling at the reporting precision.

MAT_291 describes alloy transformation mechanisms [9], so the TC-85 assignment has a phenomenological interpretation. Curve-level recovery agreement and constitutive validation address different aspects of model behaviour. The former concerns the paired recovery series; the latter concerns material response across loading and thermal histories. This distinction governs the interpretation of the numerical thermal endpoints.

### 4.4. Stress Magnitude and Numerical Uncertainty

Initial stress maxima exceed the recalculated room-temperature tensile strengths by factors of approximately 1.48 for PET-G and 1.37 for TC-85. Equivalent stress in a constrained multiaxial state and uniaxial tensile strength are different quantities, so these ratios do not demonstrate fracture. They identify conditions in which elastic continuation needs scrutiny. Effective strains near 0.16–0.20 also make geometric and material nonlinearity consequential. Yielding, residual deformation and damage require an appropriate constitutive law and local stress–strain histories. The numerical initial condition was 26.85 °C, whereas the tensile-strength records were tabulated at 23 °C.

Medium-to-fine differences in the reported results were below 5% for initial peak strain. This result is specific to the assessed outcome and loading stage. Peak stress, contact and thermal response can have different discretisation sensitivities. Boundary conditions, transition temperature and interactions between material parameters are additional sources of model uncertainty; deterministic differences between stages should be interpreted separately from probabilistic uncertainty estimates.

### 4.5. Manufacturing History and Oral Exposure

The common homogeneous geometry compares material representations while holding the nominal geometry and loading protocol constant. Fabricated appliances may also differ in thickness distribution, orientation, residual stress and defects. Print orientation and post-curing affect dimensional accuracy [2], manufacturing routes can affect fit [3], and thermoforming and ageing can alter PET-G properties [1]. The numerical contrast therefore cannot isolate manufacturing route or polymer chemistry from all processing-dependent material effects.

The specimens were conditioned in air for 48 h, and recovery was measured separately in a heated water bath. These conditions do not reproduce sustained oral-fluid exposure. Water uptake, plasticisation and ageing can alter stiffness, transition behaviour and relaxation in a material-dependent manner [8]. Conditioning may be particularly influential near the TC-85 transition. Translation to oral performance consequently depends on material conditioning, appliance geometry and tooth-level force delivery. The reported ranking is specific to the comparison conditions considered here.

### 4.6. Study Limitations

The elevated-temperature tensile–DMA discrepancy remains unresolved. The temperature-dependent constitutive description is incomplete, and the recovery comparison is not independent validation. The finite element analysis is limited to one arch, homogeneous material assignments and scalar stage maxima. Co-located stress–strain histories and independent cooling-stage verification are lacking; mesh convergence was assessed only for initial peak strain. Contact and boundary sensitivity, simultaneous parameter uncertainty, periodontal ligament, alveolar bone, oral-fluid exposure and long-term ageing were outside the analysis. These limits preclude conclusions about tooth movement, failure, cyclic durability or clinical superiority.

## 5. Conclusions

PET-G exhibited higher tensile modulus across 23–80 °C, whereas TC-85 exhibited greater benchmark recovery at 60 min. DSC rise, DMA loss-tangent maximum and early stiffness loss captured distinct thermal features. Tensile–DMA differences became more pronounced at elevated temperatures and limit a unified temperature-dependent constitutive interpretation. Under the common 600 N thermomechanical sequence, the aligner models showed lower initial strain for PET-G and a larger thermal-stage strain reduction for TC-85. Medium-to-fine mesh changes were below 5% for initial peak strain. These findings describe material contrasts and numerical responses under the specified conditions. Independent validation is needed before clinical material-selection claims can be made.

## Figures and Tables

**Table 1:** Tensile modulus (mean ± sample SD; five observations per material and temperature).

Temperature (°C)	PET-G E (MPa)	TC-85 E (MPa)
23	1396.4 ± 44.9	675.4 ± 21.2
37	1078.4 ± 41.6	463.7 ± 15.7
55	781.9 ± 22.7	269.5 ± 8.3
80	503.3 ± 17.0	127.8 ± 3.6

**Table 2:** The relationship between characterisation data and the material comparison.

Characterisation or Input	Role in the Comparison	Scope
Tensile modulus at 23 °C	Reference elastic stiffness	Experimental group statistics and model reference values
Tensile properties at 37, 55 and 80 °C	Temperature-dependent stiffness and strength comparison	Experimental temperature series
DSC and DMA series	Thermal-transition region and E′ comparison	Distinct descriptors at 5 °C reporting intervals
Shape-recovery benchmark	Assessment of the recovery response	Agreement of paired benchmark and model curves
Relaxation and creep functions	Quantitative curve descriptions in Table S5	Algebraic time dependence
Flexural properties	Supporting bending-stiffness comparison	Three-point bending at 23, 37 and 55 °C
Density, Poisson ratio and CTE	Assigned mechanical and thermal inputs	Reference material inputs in Table S7

**Table 3:** The numerical endpoints for the 600 N thermomechanical loading sequence. Strain is dimensionless. A dash denotes an unreported quantity. Full numerical precision is retained in Supplementary Data S1.

Stage and Temperature	Strain PET-G/TC-85	Displacement PET-G/TC-85 (mm)	Equivalent Stress PET-G/TC-85 (MPa)
Initial loading 300 K (26.85 °C)	0.16484/0.19567	0.1319/0.1565	80.8/47.9
Heating endpoint 352 K (78.85 °C)	0.04974/0.01487	0.0401/0.0118	15.2/1.7
Cooling endpoint 310 K (36.85 °C)	0.04974/0.01470	—	—

## Data Availability

Supplementary Data S1 contains the tensile records and group statistics, thermal series, recovery pairs, numerical endpoints and mesh data. A data dictionary specifies the source ranges and derived quantities, and the accompanying script reproduces the descriptive calculations.

## Supplementary Materials

The following supporting information can be downloaded at https://www.mdpi.com/article/10.3390/polym18202517/s1, Tables S1–S10, Figure S1 and Supplementary Data S1 with numerical datasets, source definitions and calculation procedures.
