# Supplementary material for: Thermomechanical Comparison of PET-G and TC-85 Orthodontic Aligner Materials
Source: Polymers (Basel). 2026 Oct 14;18(20):2517. doi: 10.3390/polym18202517 (PMC13708969; doi:10.3390/polym18202517)
Supplement: Supplementary file 1 [file polymers-18-02517-s001.zip › polymers-4543773-supplementary.pdf]

Supplementary Materials

Thermomechanical Comparison of PET-G and TC-85 Orthodontic Aligner Materials

Melis Büşra Aşkın, Serkan Görgülü and Gökhan Serhat Duran

These supplementary materials present the tensile and flexural properties, thermal descriptors, mesh-refinement comparisons, recovery curves, time-dependent response functions, material inputs, Vickers hardness and numerical loading schedule for the PET-G and TC-85 comparison. Experimental preparation and test settings are described in manuscript Section 2.1. Data definitions and reproducible calculations are provided in Supplementary Data S1.

S1 Tensile properties

Table S1. Tensile-property summaries (mean ± sample SD; five tabulated observations per condition).

Material	T (°C)	E (MPa)	UTS (MPa)	Elongation (%)	n
PET-G	23	1396.4 ± 44.9	54.8 ± 1.5	110.0 ± 3.1	5
PET-G	37	1078.4 ± 41.6	46.3 ± 1.8	123.9 ± 3.9	5
PET-G	55	781.9 ± 22.7	37.2 ± 1.0	146.3 ± 5.0	5
PET-G	80	503.3 ± 17.0	27.7 ± 0.9	175.3 ± 5.0	5
TC-85	23	675.4 ± 21.2	35.0 ± 0.8	178.3 ± 2.9	5
TC-85	37	463.7 ± 15.7	26.7 ± 1.0	212.6 ± 4.9	5
TC-85	55	269.5 ± 8.3	18.3 ± 0.6	262.2 ± 7.1	5
TC-85	80	127.8 ± 3.6	11.1 ± 0.4	327.4 ± 12.3	5

S2 Thermal markers and comparison of moduli

The largest sampled DSC increments occur over 75–80 °C for PET-G and 40–45 °C for TC-85. The tan δ maxima occur at 80 and 45 °C. The first 5% loss in E′ relative to 20 °C occurs within 60–65 °C and 25–30 °C. These descriptors use the 5 °C reporting grid. The DMA heating rate was 5 °C/min, and the DSC heating and cooling rates were 10 °C/min. The nominal transition estimates of 78 and 43 °C remain distinct from the sampled DSC and DMA markers.

Table S2. Descriptive comparison of tensile modulus and DMA storage modulus. Difference = 100 × (E′ − E)/E′.

Material	T (°C)	Tensile E (MPa)	DMA E′ (MPa)	Difference (%)
PET-G	23	1396.4	1399.4	0.2
PET-G	37	1078.4	1392.1	22.5
PET-G	55	781.9	1374.5	43.1
PET-G	80	503.3	579.1	13.1
TC-85	23	675.4	703.2	4.0
TC-85	37	463.7	574.8	19.3
TC-85	55	269.5	75.4	-257.5
TC-85	80	127.8	43.7	-192.5

E′ at 23 and 37 °C was linearly interpolated from adjacent DMA entries. E′ at 55 and 80 °C is directly tabulated. The between-method comparison uses matched nominal temperatures. A negative difference indicates that the tensile modulus exceeds E′.

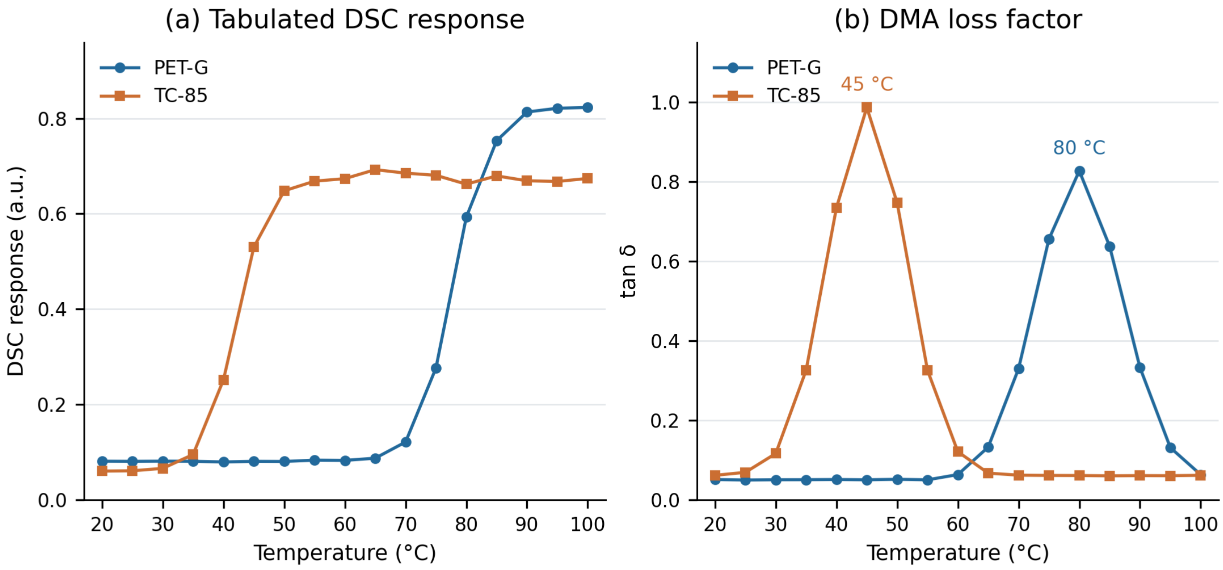

Figure S1. Thermal response series. (A) DSC signal in arbitrary units. (B) DMA tan δ. Markers preserve the 5 °C spacing; lines are visual guides. The sampled DSC rise and DMA peak are distinct thermal descriptors.

S3 Mesh refinement

Table S3. Peak effective strain at 600 N and 300 K. Changes use the next-finer result as the denominator.

Mesh	Elements	PET-G strain	TC-85 strain	Change to finer PET-G / TC-85 (%)
Coarse	119,369	0.155777	0.180947	3.79 / 5.04
Medium	296,750	0.161912	0.190557	1.78 / 2.61
Fine	671,671	0.164839	0.195673	—

The coarse-to-medium TC-85 difference exceeds the 5% criterion. Both medium-to-fine differences are below 5%. The criterion applies to the peak effective strain at the initial 600 N and 300 K condition.

S4 Recovery series agreement

Recovery specimens were 50 × 10 × 1.0 mm strips bent around a 4 mm diameter mandrel at 80 °C for 5 min, cooled under constraint to 24 °C, held for 5 min, then fully unloaded. Free recovery was recorded by video for 60 min in an 80 °C water bath.

Table S4. Recovery fractions used to calculate curve agreement. Values are rounded for display; Supplementary Data S1 provides the full-precision values.

Time (min)	PET-G benchmark	PET-G model	TC-85 benchmark	TC-85 model
0	0.0000	0.0000	0.0000	0.0000
5	0.1022	0.1178	0.2977	0.3169
10	0.2173	0.2143	0.5161	0.5258
20	0.3852	0.3579	0.7436	0.7543
30	0.4861	0.4542	0.8465	0.8537
45	0.5909	0.5426	0.9131	0.9081
60	0.6333	0.5910	0.9254	0.9237

MAE and RMSE are calculated from all seven pairs, including time zero, using unrounded values. PET-G MAE = 0.0240 and RMSE = 0.0296; TC-85 MAE = 0.0076 and RMSE = 0.0097. The percentage-point equivalents are 2.40/2.96 and 0.76/0.97. These metrics describe agreement

between the benchmark and model curves. As the benchmark informed the recovery response, this comparison is not held-out validation.

S5 Time-dependent response descriptors

Table S5 gives the algebraic functions for normalised residual force, creep strain and recovery fraction, with t in minutes. The PET-G/TC-85 relaxation constants are 15/20 min (900/1200 s), the creep constants are 22/16 min and the recovery constants are 25/12 min. These are coefficients of the tabulated response functions; they do not specify the complete temperature-dependent solver material law.

Table S5. Algebraic descriptions of time-dependent response.

Quantity	PET-G	TC-85
Normalised residual force	$0.45 + 0.55 \exp(-t/15)$	$0.62 + 0.38 \exp(-t/20)$
Creep strain	$0.040 + 0.035[1 - \exp(-t/22)]$	$0.050 + 0.055[1 - \exp(-t/16)]$
Recovery fraction	$0.65[1 - \exp(-t/25)]$	$0.93[1 - \exp(-t/12)]$

The recovery coefficients 0.65 and 0.93 denote asymptotic fractions. At 60 min, the functions give approximately 0.5910 and 0.9237; the corresponding benchmark fractions are 0.6333 and 0.9254. Recovery fractions and aligner strain maxima are analysed as separate response measures.

S6 Flexural summary

Table S6. Tabulated flexural modulus summaries.

Temperature (°C)	PET-G flexural modulus (MPa)	TC-85 flexural modulus (MPa)
23	1550	760
37	1180	520
55	720	240

The three-point-bending tests used 30 × 10 × 1.0 mm strips, a 16 mm support span and a crosshead speed of 1 mm/min at 23, 37 and 55 °C. The flexural values describe a different loading configuration from tension and DMA.

S7 Reference material inputs

Table S7. Reference properties and model inputs. Elastic modulus was informed by room-temperature tensile characterisation; density, reference Poisson ratio and CTE were assigned values.

Input	PET-G	TC-85
Elastic modulus (MPa)	1396	675
Poisson ratio	0.36	0.40
Density (g cm^{-3})	1.27	1.15
Thermal expansion coefficient (K^{-1})	6.5×10^{-5}	1.0×10^{-4}
Nominal transition reference (°C)	78	43

The modulus inputs are the reference values associated with the numerical comparison. Experimental means are reported in Tables 1 and S1. Thermal expansion was referenced to 300 K for both materials. The nominal transition references remain distinct from the DSC and DMA descriptors in section S2. Additional constitutive coefficients are listed in Table S9.

S8 Vickers microhardness

Table S8. Vickers hardness (mean ± sample SD of five indentations per material group).

Material	Vickers hardness (HV)	Indentations
PET-G	12.04 ± 0.35	5
TC-85	8.19 ± 0.12	5

Hardness was measured on 10 × 10 × 1.0 mm flat coupons at 23 °C using a Qness 60 M Evo, a 1.96 N load and a 10 s dwell time (HV0.2). The five tabulated values per material represent indentations. Hardness measures resistance to local indentation and is not directly interchangeable with tensile or flexural modulus.

S9 Constitutive coefficients and numerical sequence

Table S9. Additional material-model coefficients. MAT_291 symbols retain their solver definitions; EM/EA are martensite/austenite branch moduli in the alloy formulation, used here as phenomenological parameters for TC-85.

Material	Coefficient	Value
PET-G	Shear-relaxation fraction g_1	0.55
PET-G	Equilibrium fraction	0.45

Material	Coefficient	Value
PET-G	Arrhenius activation energy	50 kJ/mol
TC-85	EM / EA	675 / 127.8 MPa
TC-85	PRM / PRA	0.40 / 0.40
TC-85	CPM / CPA	1.725 / 1.725 MPa/K
TC-85	LH	0.56907 MPa

The PET-G representation used finite-strain thermoviscoelasticity. TC-85 used symmetric isotropic transformation surfaces within the phenomenological MAT_291 assignment. CPM and CPA are volumetric heat capacities; LH is latent heat per unit volume. The alloy branch names do not imply physical martensitic or austenitic phases in the polymer.

Table S10. The prescribed load and temperature sequence. The compressive load was maintained at 600 N after the initial ramp.

Stage	Time interval (s)	Load (N)	Temperature (K)
Loading ramp	0–10	0 to 600	300
Initial hold	10–60	600	300
Heating ramp	60–660	600	300 to 352
Heating hold	660–1260	600	352
Cooling ramp	1260–1860	600	352 to 310
Final hold	1860–2460	600	310

Finite-sliding separable surface contact used $\mu s = \mu d = 0.20$. A 2 mm posterior support band was defined in the terminal molar regions, and the distributed load resultant was (0, 0, −600) N. Nonlinear implicit dynamics used 10-node quadratic tetrahedral elements (ELFORM 16), at least three layers through the thickness, an initial time step of 0.01 s, a minimum of 10^{-4} s and upper limits of 0.5–5 s. The Newmark parameters were $\gamma = 0.55$ and $\beta = 0.275625$.

S10 Supplementary Data S1

Supplementary Data S1 provides numerical values, variable and unit definitions, source selections and a Python script for the descriptive calculations. Full-precision values are retained for calculation; rounding is applied only to displayed summaries.
